# *SiMYB56* Confers Drought Stress Tolerance in Transgenic Rice by Regulating Lignin Biosynthesis and ABA Signaling Pathway

**DOI:** 10.3389/fpls.2020.00785

**Published:** 2020-06-18

**Authors:** Weiya Xu, Wensi Tang, Chunxiao Wang, Linhao Ge, Jianchang Sun, Xin Qi, Zhang He, Yongbin Zhou, Jun Chen, Zhaoshi Xu, You-Zhi Ma, Ming Chen

**Affiliations:** ^1^National Key Facility for Crop Genetic Resources and Genetic Improvement, Key Laboratory of Crop Genetics and Breeding, Ministry of Agriculture, Institute of Crop Science, Chinese Academy of Agricultural Sciences, Beijing, China; ^2^Institute of Crop Sciences, Ningxia Academy of Agriculture and Forestry Sciences, Yongning, China

**Keywords:** foxtail millet, R2R3-MYB transcription factor, drought tolerance, lignin biosynthesis, ABA signaling pathway

## Abstract

Foxtail millet (*Setaria italica*) originated in China and is generally cultivated in arid and barren soil. Through long-term harsh environmental selection, foxtail millet has acquired significant drought resistance. However, the molecular mechanism of foxtail millet drought resistance is still unknown. Here, we identified a drought-induced R2R3-MYB transcription factor *SiMYB56* in foxtail millet. Overexpression of *SiMYB56* significantly enhances tolerance to drought stress in transgenic rice plants at both the vegetative and the reproductive stage and has no adverse effect on its normal growth. Compared with wild-type controls, *SiMYB56*-overexpressing rice plants had lower MDA content and higher lignin content under drought conditions. Quantitative real-time PCR and Transcriptional activity assays demonstrated that *SiMYB56* could activate expression of lignin biosynthesis genes under drought conditions. Also, we found that overexpression of *SiMYB56* can led to ABA accumulation in the seeds transgenic rice plants. Further experiments showed that Overexpression of *SiMYB56* can upregulate the expression of ABA synthesis and response related genes under drought conditions. In conclusion, *SiMYB56* may enhance the drought resistance of transgenic rice plants by regulating lignin biosynthesis and ABA signaling pathway, making *SiMYB56* a candidate gene for drought resistance improvement in gramineous crops.

## Introduction

Drought is a major environmental factor that affects the natural geographical distribution of plants, limits agricultural plant productivity, and threatens food security (Lesk et al., 2016). As a consequence of drought stress, plants suffer photosynthesis inhibition, metabolic dysfunction, and cellular structure damage (Xu et al., 2010). Foxtail millet (*Setaria italica*), originated from China, is an important food and fodder grain crop in arid and semi-arid regions of Asia and Africa (Barton et al., 2009; Lu et al., 2009). Small diploid genome size (∼510 Mb), self-pollination, low repetitive DNA content (30%) and short life cycle, making it an ideal and suitable model species for genetic and molecular studies (Doust et al., 2009; Diao et al., 2014; Muthamilarasan and Prasad, 2015). Also, foxtail millet is an elite drought-tolerant crop, water use efficiency of foxtail millet has been shown to be higher than that of maize, wheat, and sorghum (Gu et al., 1987). Because of its excellent drought tolerance and water-use efficiency, research on the mechanisms of drought tolerance of foxtail millet have great significance for crop drought-resistance molecular breeding. However, the molecular mechanism of its drought adaptation is still not clear.

Lignin biosynthesis plays an important role in plant biotic stress resistance, especially in disease resistance (Li et al., 2020), and insect resistance (Wang et al., 2017). In recent years, the role of lignin in plant response to abiotic stress has been gradually revealed. Studies have found that high levels of lignification in plants can lead to increased drought tolerance, although the associated regulatory networks have not been well elucidated (Hu et al., 2009; Pereira et al., 2018). It is widely accepted that lignin biosynthesis is controlled by a multi-leveled hierarchical regulatory network involving NAC and MYB transcription factors (TFs) (Zhao and Dixon, 2011; Ohtani and Demura, 2019). Some NAC TFs, including *VND1-7* and *NST1-3*, were found to serve as primary switches of this network (Kubo et al., 2005; Mitsuda et al., 2007). *MYB46* and *MYB83*, the downstream targets of NAC proteins, are secondary switches of this network (McCarthy et al., 2009). Further downstream are some other MYB genes implicated in lignin biosynthesis regulation (Ko et al., 2014). Although a battery of R2R3-MYB TFs have been identified to be involved in lignin biosynthesis in model plants like *Arabidopsis*, the mechanism of lignin regulation in major crops, especially Gramineae, remains unknown.

Abscisic acid (ABA), which is the central regulator of abiotic stress resistance in plants, coordinates an array of functions enabling plants to cope with different stresses (Sah et al., 2016). Abiotic stresses, especially water deficit, induce ABA accumulation, which triggers rapid biochemical and physiological responses that enhance stress adaptation (Finkelstein, 2013; Ng et al., 2014). As the largest subfamily of MYB transcription factor family, numerous evidences have shown that R2R3-MYB transcription factors play important roles in ABA signaling under drought stress (Shinozaki and Yamaguchi-Shinozaki, 2007; Baldoni et al., 2015). In Arabidopsis, *AtMYB2* increased drought resistance by activating the expression of related genes induced by ABA under drought stress (Abe et al., 2003). *AtMYB96* improves the drought resistance of transgenic Arabidopsis through activating cuticular wax biosynthesis in an ABA-dependent way (Seo et al., 2011). However, most identified R2R3-MYB transcription factors involved in ABA mediated plant drought resistance play roles in ABA signal transduction, and few of them are found play roles in ABA synthesis.

It has been pointed out that there are at least 209 MYB transcription factors in foxtail millet, and 68% of them function in response to stress (Muthamilarasan et al., 2014). However, no further research has been done on these transcription factors except for *SiMYB3* which mediates the low nitrogen tolerance of foxtail millet (Ge et al., 2019). Here, we identified a drought-induced R2R3-MYB transcription factor in foxtail millet *SiMYB56*. Overexpression of *SiMYB56* in rice significantly improved the drought resistance of transgenic rice throughout their entire growing season. By analyzing the regulation mechanism, we found that *SiMYB56* improved the drought resistance of transgenic rice by regulating lignin biosynthesis and ABA signaling pathway, which enriches the study of functional genomics of drought resistance in foxtail millet and provides new strategies for improving the drought resistance of gramineous crops.

## Materials and Methods

### Plant Materials, Growth Conditions, and Stress Treatments

For analysis of *SiMYB56* expression under different stress treatments, foxtail millet (Yugu1) seeds were soaked in water, germinated at 28°C for 2 days, and then transferred to Hoagland solution for 2 weeks in a growth chamber (60% relative humidity) under a 14-hour-light (21°C)/10-h-dark (24°C) cycle. Next, 2-week-old seedings were exposed to various abiotic stresses, including osmotic stress (10% PEG6000), salinity stress (80 mM NaCl) and ABA presence (100 μM ABA). Leaves, stems, and roots were sampled at 0, 1, 3, 6, 12, and 24 h, and all samples were immediately frozen in liquid nitrogen and stored at −80°C prior to analysis.

For phenotypic analysis of transgenic rice plants and wild-type controls under drought stress during vegetative stage, we carried out soil drought and hydroponic drought experiments. For the soil drought experiments, germinated seeds of wild-type controls (Ki) and transgenic rice plants (OE16/OE21/OE30) were transferred to soil. Then, rice seedlings were grown under a 14-hour-light (30°C)/10-hour-dark (26°C) cycle in the greenhouse. At the 5-leaf stage, watering was stopped until all wide-type controls wilted. Then, plants were re-watered for 7 days. After re-watering, survival rate statistics were collected.

For the hydroponic drought experiments, we treated wild-type controls and transgenic rice plants with 10% PEG6000 to simulate osmotic stress caused by drought. Uniformly germinated seeds were sown into a 96-well plate that had its bottom removed. The plate was floated on water for one week and then transferred to Hoagland solution for another one week. For PEG treatment, 2-week-old seedlings were transferred to culture solution containing 10% PEG6000 and incubated for another 2 weeks before physiological indexes were measured.

For phenotypic analysis of transgenic rice plants and wild-type controls under drought stress during reproductive stage, we carried out field-drought resistance experiments. In order to achieve a better effect from field drought, we chose to carry out the experiment in Ningxia province, which receives relatively little rain. Three independent T3 homozygous transgenic rice lines (OE16, OE21, and OE30), alongside wild-type controls (Ki), were transplanted to a paddy field at the Wanghong experimental station, Ningxia Academy of Agriculture and Forestry Sciences (Ningxia, China). A randomized design was employed with two replicates (2017) or three replicates (2018). At 30 days after sowing, seedlings were randomly transplanted with 15- × 30-cm spacing and a single seeding per hill. Each line was in four 1.2 m long rows. One week after transplanted seedlings were established, irrigation was withheld for the rest of the growing season. When the rice matured, agronomic traits were determined.

### RNA Isolation and Quantitative Real-Time PCR

Total RNA was extracted from seedlings using the Total RNA Extraction Kit (TIANGEN, China). The cDNA was synthesized in accordance with Fast Quant RT Super Mix Reverse Transcription Kit instructions (TransGene, Beijing, China). Real-time PCR amplification was performed using a Real Master Mix (SYBR Green, Beijing, China) kit (TransGene) and a fluorescence quantitative PCR instrument (ABI7500, United States). The relative gene expression in different samples was calculated using the 2^–ΔΔCt^ method, using the Ct value at the specific fluorescence threshold for each sample. The actin genes for foxtail millet (Si001873m.g) and rice (LOC_Os03g50885) were used as internal controls. Quantitative real-time PCR was performed in triplicate. Primers used for quantitative real-time PCR are listed in Supplementary Table S1.

### Subcellular Localization Assay

The full-length cDNA of *SiMYB56* without stop codon were amplified and cloned into the BamHI site of vector 16318hGFP. The resulting SiMYB56-GFP fusion construct, driven by a CaMV35S promotor, and the positive control 16318hGFP were separately transformed into Arabidopsis mesophyll protoplasts using a PEG-calcium mediated method. This was followed by a 12–24 h incubation to allow transient expression. Chlorophyll autofluorescence and H2B-mCherry were used as chloroplast and nuclear markers, respectively. The fluorescence in protoplast cells was visualized using a confocal microscope (Zeiss LSM700, Carl Zeiss, Oberkochen, Germany) and images were acquired with ZEN 2010 software (Carl Zeiss, Oberkochen, Germany). Arabidopsis protoplast cells were prepared based on a previous report (Yoo et al., 2007). Primers used are listed in Supplementary Table S1.

### Transcription Activation Assay in Yeast

The *SiMYB56* CDS sequence was amplified by PCR and fused in-frame with the GAL4 DNA binding domain by cloning into the NdeI site of vector pGBKT7 (Clontech). The constructs pGBKT7-SiMYB56 and the negative control pGBKT7 were separately transformed into yeast strain AH109 *Saccharomyces cerevisiae* through LiAc-mediated transformation according to the manufacturer’s instructions (Clontech). Transformants was cultured on SD/–Trp medium at 28°C. After 2 days, the positive transformants were plated on SD/–Trp and SD/–Trp/–His/–Ade medium, respectively. The transcriptional activation activities were evaluated according to their growth status. Primers used are listed in Supplementary Table S1.

### Transcriptional Repression Assay in Protoplasts

Transcriptional repression analysis was performed in protoplasts as described previously (Liu P. et al., 2018), For effectors vector, the coding sequences of *SiMYB56* were first cloned into pGBKT7. Then the coding regions of BD-SiMYB56 fusion and BD were separately amplified and cloned into the PstI site of vector pGreenII 62-SK to get the effector plasmids (35S:BD-SiMYB56, 35S:BD). For the reporter vectors, the vector containing the firefly luciferase (LUC) reporter gene driven by the minimal TATA box plus five GAL4 binding elements and CaMV 35S promoter was used for transcriptional repression test. The Renilla luciferase gene driven by the CaMV 35S promoter, was used as an internal control. The internal control, effector and reporter were simultaneously transformed into the protoplast cells, then kept in dark for 16 h. The activities of LUC and REN were separately determined using Dual-Luciferase Reporter Assay System (Promega, E1910). Primers used are listed in Supplementary Table S1.

### Sequence Alignment, Phylogenetic, and Promoter *Cis*-Acting Element Analyses

To investigate the relationship between *SiMYB56* and other MYB transcription factors from Arabidopsis and rice, amino acid sequences were compared using DNAMAN software, and a systematic phylogenetic analysis was carried out using the neighbor-joining method in MEGA7.0 software with 1000 bootstrap replications. Amino acid sequences for Arabidopsis MYB TFs were acquired from TAIR^1^ and those for rice MYB TFs were acquired from the Phytozome database^2^. For *cis*-element analysis, 2000 bp upstream of the translational start codon were extracted from Phytozome and examined as the promoter region. Potential promoter *cis*-elements for each gene were identified using the PlantCARE database^3^.

### Transcriptional Activity Assays in *Nicotiana benthamiana*

Transcriptional activity assays were performed in *Nicotiana benthamiana* leaves with reference to the methods described previously (Liu et al., 2017). The 2-kb promoters for *4CL5* and *F5H1* were fused with the luciferase reporter gene LUC in vector pGreenII0800 to generate reporter constructs 4CL5_pro_: LUC and F5H1_pro_: LUC, respectively. Full-length *SiMYB56* was separately cloned into the plant binary vector pCAMBIA1302 to generate the effector construct 35S:SiMYB56-GFP. The reporter and effector constructs were separately introduced into *Agrobacterium* strain GV3101(pSoup-p19), to carry out the co-infiltration (1:1) in *Nicotiana benthamiana* leaves. the LUC activities were observed and analyzed 48 h after infiltration using the Night SHADE LB 985 system (Berthold, Germany). After luciferin (100 μM) spraying, leaves were kept in the dark for 5 min, and then fluorescence was observed. Primers used are listed in Supplementary Table S1.

### Plasmid Construction and Rice Transformation

The *SiMYB56* coding region was amplified by PCR using the primers pMWB014-SiMYB56-F and pMWB014-SiMYB56-F. The PCR product was then ligated into the binary vector pMWB014 (Digested by BamHI) to obtain the construct Ubi:SiMYB56-FLAG. This construct was transformed into Oryza sativa cv. Kitaake using *Agrobacterium*-mediated transformation (Hiei et al., 1994). Seventeen transgenic rice lines were generated and confirmed by PCR using primers Test-F (targeting the ubiquitin promoter) and Test-R (targeting the NOS terminator sequence). three transgenic lines (OE16, OE21, and OE30) were analyzed for phenotypes. Primers used are listed in Supplementary Table S1.

### Germination Assays

For the germination assay, about 200 seeds from each of the transgenic and wild-type rice seeds were surface-sterilized in 10% (v/v) NaClO for 15 min, followed by washing five times with sterilized distilled water. Next, the transgenic and wild-type rice seeds (16 seeds, respectively) were evenly placed on the four corners of a plate covered with two layers of filter paper and then 0, 2.5, or 5 μM ABA solution were add to the plate, respectively. The percentage of germinated seeds was calculated 2 days later. Each experiment included three replicates. One week later, germinated seed sprouts were sampled and frozen in liquid nitrogen for subsequent ABA content determination.

### Measurements of the ABA Content, MDA Content, Lignin Content, and Relative Electrolyte Leakage

Measurements of ABA content: One-week-old sprouts (0.1 g) of transgenic and wild-type rice seeds germinated under the 0 μM ABA treatment described above were extracted to measure ABA content. The experiments were performed in accordance with protocols for the Plant ABA ELISA Kit (Jianglaibio, JL13378-48T, Beijing, China). Each experiment included three replicates.

Measurements of MDA content: Leaves of 4-week-old seedlings (0.1 g) of transgenic and wild-type rice plants, grown under normal and 2-week 10% PEG6000 treatments described above, were extracted to measure MDA content. The experiments were performed in accordance with MDA assay kit protocols (Comin, MDA-1-Y, China). Each experiment included three replicates.

Measurements of lignin content: Leaves of 4-week-old seedlings (0.1 g) of transgenic and wild-type rice plants grown under normal and 2-week 10% PEG6000 treatments described above, were extracted to measure Lignin content. The experiments were performed following Lignin ELISA Kit protocols (Jianglaibio, JL22761-48T, Beijing, China). Each experiment included three replicates.

Measurements of relative electrolyte leakage: Leaves of 4-week-old seedlings (0.1 g) of transgenic and wild-type rice plants grown under normal and 2-week 10% PEG6000 treatments described above, were extracted to measure relative electrolyte leakage. The relative electrolyte leakage was examined in accordance with methods described in Cao et al. (2007).

### Histochemical Staining

Hand-cut sections of 2-week-old leaves of transgenic rice plants and wild-type controls under normal condition or 3-week-old leaves of transgenic rice plants and wild-type controls under 10% PEG6000 treatment for about one week were soaked in an ethanol solution containing 1% (w/v) phloroglucinol for 2 min, and were then immersed in concentrated HCl for another 2 min (Herrera-Ubaldo and de Folter, 2018). The stained sections were then examined using a Leica DMRB microscope (Leica, Wetzlar, Germany).

## Results

### *SiMYB56* Belongs to Subgroup 21 of the R2R3-MYB Transcription Factor Family

**FIGURE 1:**
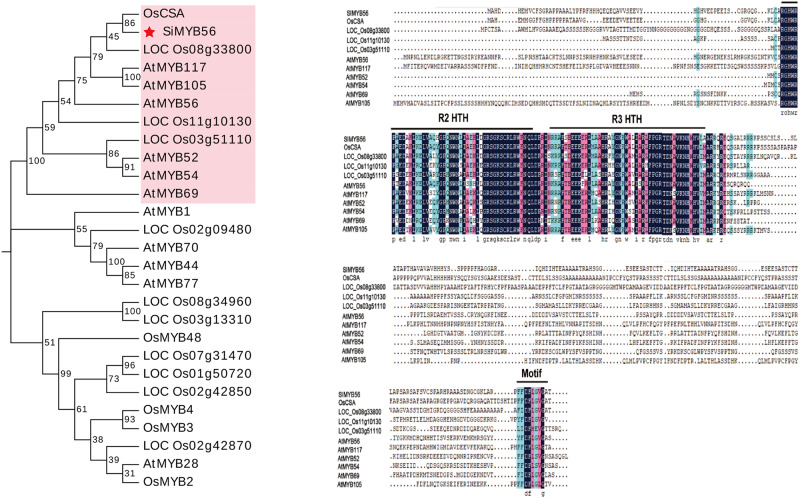
Phylogenic and molecular analyses of *SiMYB56*. The filled red star indicates *SiMYB56*, and genes which are clustered in the same phylogenetic tree branch as *SiMYB56* are labeled in pink. Two domains putatively responsible for the R2R3-MYB structure and a motif that belongs to *Arabidopsis* R2R2-MYB subgroup 21 are indicated. Identical amino acids are highlighted in blue.

*SiMYB56* is a 1296 bp long gene that encodes a putative 322 amino acid protein with a calculated molecular mass of 34.9 kD and a pI of 8.31 (Supplementary Figure S1A) and is an R2R3-MYB transcription factor (Supplementary Figure S1B). Phylogenic and molecular analyses showed that *SiMYB56* belongs to R2R3-MYB transcription factor subgroup 21 and is most closely related to rice *CSA* (*Carbon Starved Anther*) (Figure 1).

### Expression of *SiMYB56* Can Be Induced by Various Abiotic Stresses

**FIGURE 2:**
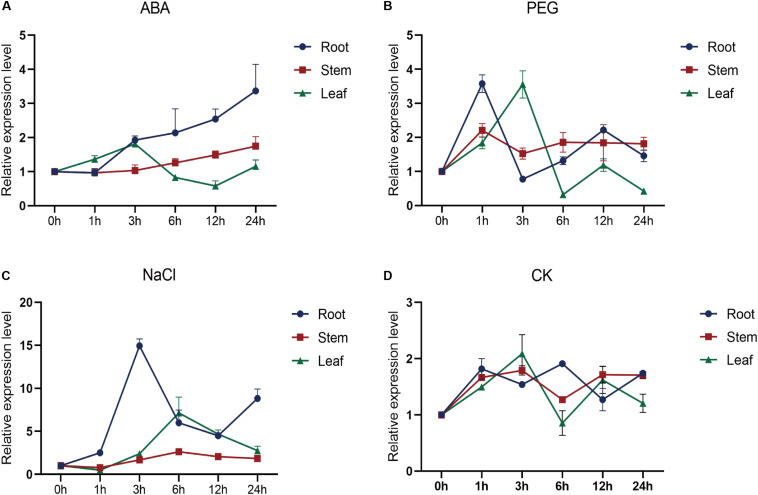
Expression profile of *SiMYB56*. Relative *SiMYB56* expression in foxtail millet under different treatments: **(A)** 100 μM ABA, **(B)** 10% PEG6000, **(C)** 80 mM NaCl, and **(D)** Normal condition. All *SiMYB56* expression levels were determined by qRT-PCR. Values shown are means ± SD (*n* = 3).

To investigate the expression profile of *SiMYB56*, plantCARE was used to analyze its promoter region for putative regulatory elements. The analysis identified several *cis*-acting promoter elements that are related to drought resistance, salt tolerance, and ABA response, such as ABRE (Kaplan et al., 2006), DRE (Dubouzet et al., 2003), G-box, and W-box (Shen and Ho, 1995; Liu et al., 2016; Supplementary Figure S2). Quantitative real-time PCR showed that *SiMYB56* expression was highest in the stem, lowest in the root (Supplementary Figure S3) and could be induced by various stresses (Figure 2A). Under ABA treatment, *SiMYB56* transcript was induced in the root, increasing gradually with time (Figures 2A,D). Under PEG6000 stress, *SiMYB56* transcript increased, peaking after a 1 h treatment in root and a 3 h treatment in leaf (Figures 2B,D). *SiMYB56* transcript was strongly induced in root and leaf under NaCl treatment, increasing about 15-fold in root after a 3 h treatment and 5-fold in leaf after a 6 h treatment (Figures 2C,D). The results of *SiMYB56* expression profile analyses were consistent with promoter element predictions, indicating that *SiMYB56* could indeed responds to various abiotic stresses in foxtail millet.

### SiMYB56 Protein Localizes to the Nucleus and Has Transcription Repression Activity

**FIGURE 3:**
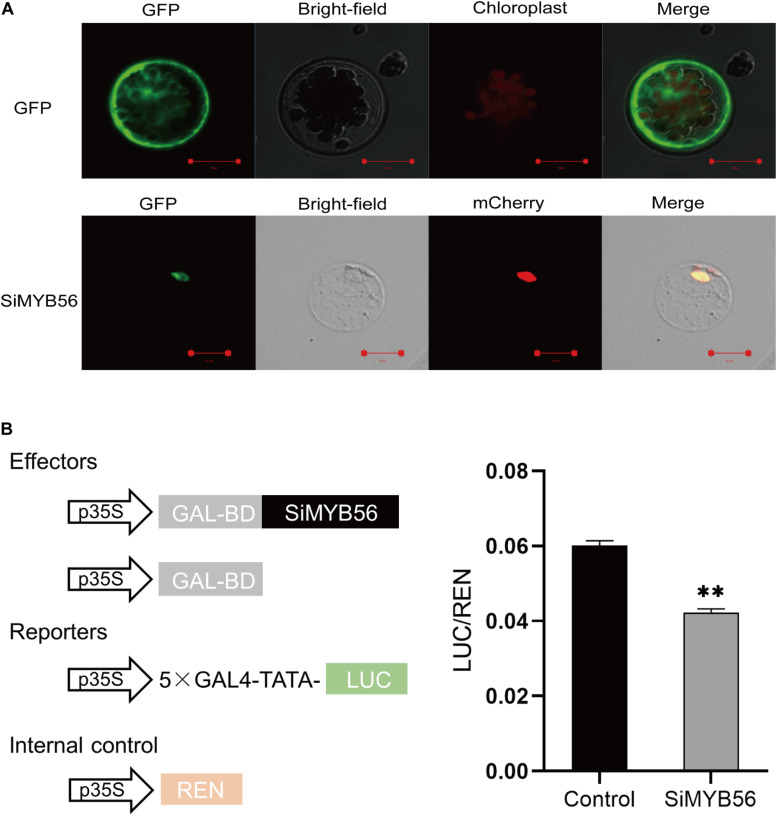
Subcellular localization and transcription repression activity of SiMYB56. **(A)** Subcellular localization of SiMYB56 protein in Arabidopsis protoplasts. The 35S:SiMYB56-GFP and 35S:GFP control vectors were transiently expressed in Arabidopsis protoplasts, separately. Nuclear marker protein (H2B-mCherry) was co-expressed with 35S:SiMYB56-GFP. Fluorescence was observed using a Zeiss LSM 710 confocal microscope 16 h after transformation. **(B)** Transcription repression activity of SiMYB56 protein in Arabidopsis protoplasts. The activities of firefly luciferase (LUC) and renilla luciferase (REN) were determined 16 h post-transformation. The relative luciferase activities in control and SiMYB56-expressed samples were calculated by normalizing the LUC values against REN. Data represent the mean ± SD (*n* = 4), **P ≤ 0.01, *t*-test.

To determine the subcellular localization of SiMYB56 protein, we generated SiMYB56-GFP fusion constructs controlled by the CaMV35S promoter. These constructs were then expressed in *Arabidopsis* mesophyll protoplasts, and plasmid 16318hGFP was used as a positive control. The results revealed that SiMYB56 fusion protein was expressed in the nucleus (Figure 3A), as confirmed by co-localization with a nuclear marker.

To test whether SiMYB56 protein had transcription activation or repression activity, the sequence of *SiMYB56* was first fused in-frame to the GAL4 DNA-binding domain in the pGBKT7 vector, and the fusion construct pBD-SiMYB56 was transformed into yeast strain AH109. Plasmid pGBKT7 served as a negative control. Both pBD-SiMYB56 and pGBKT7 transformants grew on SD/-Trp but did not grow on SD/-Trp/-Ade/-His (Supplementary Figure S4), indicating that SiMYB56 protein has no transcription activation activity. Then transcriptional repression assay in protoplasts was carried out to verify whether SiMYB56 protein has transcriptional repression activity. In this assay, the firefly luciferase (LUC) gene was fused to a 5× GAL4 binding site to generate the reporter, and the renilla luciferase (REN) gene driven by 35S promoter was used as the internal control. Meanwhile, the effector plasmid was constructed by fusing the SiMYB56 coding sequence to the GAL4 DNA binding domain (BD). Bioluminescence determination revealed that the expression of SiMYB56 led to significant obvious down-regulation of the relative luciferase activity, compared to the control (Figure 3B). These results indicate that SiMYB56 has transcription repression activity.

### *SiMYB56* Overexpression Significantly Improves Drought Tolerance of Transgenic Rice During the Vegetative Stage

**FIGURE 4:**
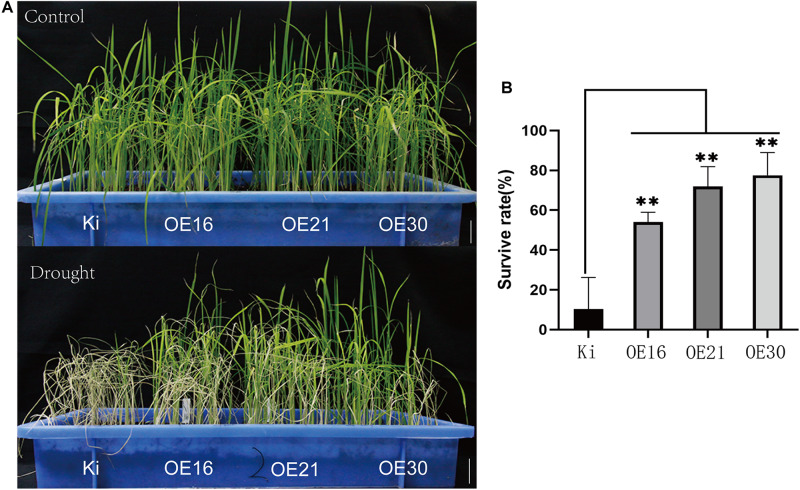
Phenotypic analysis of transgenic rice plants and wild-type controls under drought conditions. **(A)** Growth of transgenic and wild-type rice seedlings under normal and drought conditions in a soil drought experiment. Bar = 5 cm **(B)** Survival rate of wild-type and transgenic rice plants after drought stress. Data represent the mean ± SD (*n* = 4), **P ≤ 0.01, *t*-test.

To evaluate *SiMYB56* functions, drought stress tolerance analysis was carried out using homozygous T3 seeds of three transgenic rice lines (OE16/OE21/OE30) and wild-type rice (KITAKKE, Ki). Germinated seeds of homozygous transgenic rice were planted alongside germinated wild-type seeds. Two weeks later, irrigation was stopped for 15 days to subject rice plants to drought treatment. Then, the survival rate was calculated after allowing recovery following re-watering. The results showed no obvious differences in growth performance between transgenic rice plants and wild-type controls under normal growth conditions however, under drought treatments, the survival rate was significantly higher in three transgenic lines (50–80%) than in wild-type rice (10%) (Figures 4A,B), which suggested that *SiMYB56* conferred drought stress tolerance in rice during the vegetative stage.

**FIGURE 5:**
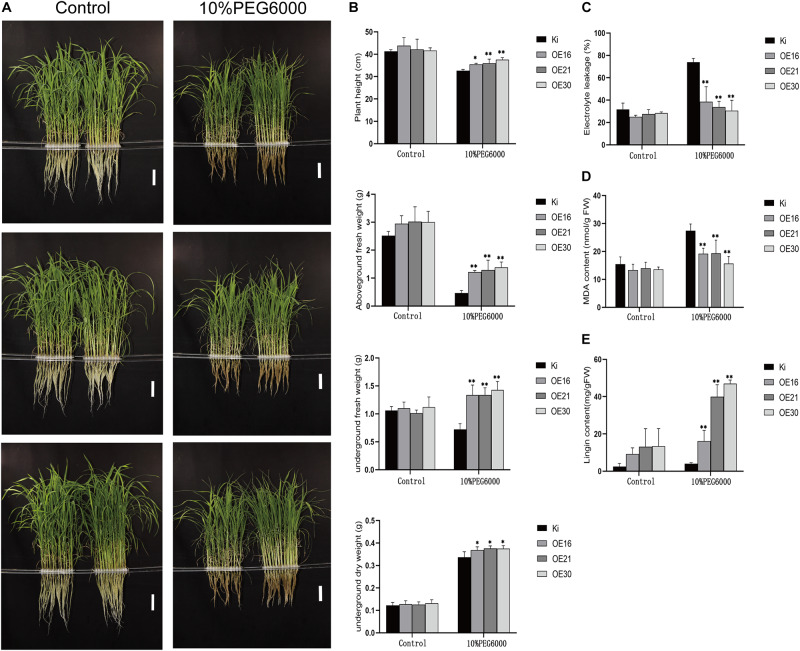
Phenotypic analysis of transgenic rice plants and wild-type controls in response to 10% PEG6000 treatments. **(A)** Phenotype of wild-type controls and transgenic rice plants under normal and 10% PEG6000 conditions in a hydroponic drought experiment. Bar = 7 cm. **(B)** Root length, plant height, aboveground fresh weight, underground fresh weight, aboveground dry weight, and underground dry weight under normal growth conditions and after 10% PEG6000 treatment. Relative electrolyte leakage **(C)**, MDA content **(D)**, and lignin content **(E)** under normal growth conditions and after 10% PEG6000 treatment. Data represent the mean ± SD (*n* = 4), *P ≤ 0.05, **P ≤ 0.01, *t*-test.

To further explore *SiMYB56* functions in rice drought resistance, we performed another stress tolerance assay using 10% PEG6000 to simulate drought stress (Figure 5A). Results showed that under 10% PEG6000 treatment, root length, plant height, aboveground fresh weight, underground fresh weight, aboveground dry weight, and underground dry weight of transgenic rice plants were significantly higher than those of wild-type controls (Figure 5B). Results of electrolyte leakage and MDA content analyses showed that, under 10% PEG6000 treatment conditions, transgenic rice plants had significantly lower electrolyte leakage and MDA content than wild-type controls, which indicated a lower degree of membrane damage in transgenic lines (Figures 5C,D). Phylogenic analyses have shown that *SiMYB56* belongs to R2R3-MYB transcription factor subgroup 21. Many members of transcription factor subgroup 21 have been reported to be involved in the synthesis of secondary cell walls (Zhong et al., 2008; Zhang et al., 2012). Lignin, one of the main components of plant secondary cell walls, is closely related to plant drought resistance (Liu Q. et al., 2018). Therefore, lignin content of transgenic rice plants and wild-type controls was then analyzed, and the results showed that transgenic rice plants had significantly higher lignin content than wild-type controls under 10% PEG6000 treatment conditions (Figure 5E and Supplementary Figure S5). These results demonstrate that *SiMYB56* may improve transgenic rice drought resistance during the vegetative stage by increasing lignin biosynthesis and reducing membrane damage.

### *SiMYB56* Overexpression Increases Transgenic Rice Yield Under Field-Drought Conditions

**FIGURE 6:**
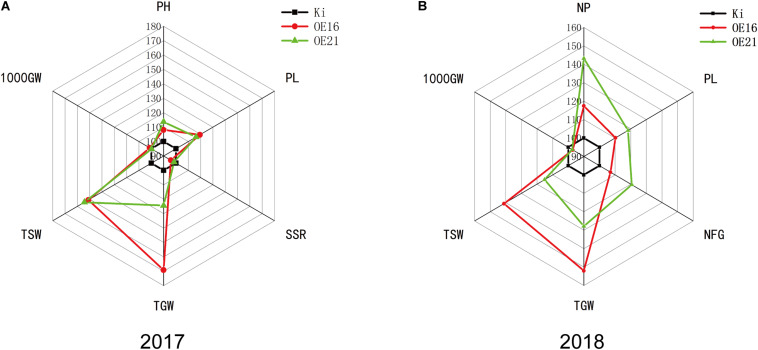
Phenotypic analysis of transgenic rice plants and wild-type controls grown under field-drought conditions. Spider plots of the agronomic traits of two independent homozygous T3 lines of transgenic rice plants and corresponding wild-type controls under field-drought conditions in year 2017 **(A)** and 2018 **(B)**. Each data point represents the percentage of the mean values (2017, *n* = 2; 2018, *n* = 3). The mean measurements from the wild-type controls were assigned a 100% reference value. PH, plant height; SSR, seed setting rate; NP, number of panicles per hill; PL, panicle length; NFG, number of filled grains; TGW, total grain weight; TSW, total straw weight; 1000GW, 1,000 grain weight.

Crop yield under drought condition is closely related to the drought resistance of crops during the reproductive stage (Boyer, 1982). Drought resistance experiments with seedlings proved that *SiMYB56* conferred drought resistance to transgenic lines during the vegetative stage. In order to study *SiMYB56* function during the reproductive stage, drought resistance experiments were carried out in the field in year 2017 and year 2018. After harvesting, agronomic traits of transgenic rice plants and wild-type controls grown under drought conditions were measured which included plant height (PH), seed setting rate (SSR), the number of panicles per plant (NP), panicle length (PL), number of filled grains (NFG), total grain weight (TGW), total straw weight (TSW), and 1000-grain weight (1000GW). Results showed that transgenic rice plants had higher total grain weight than wild-type rice under field-drought conditions both in year 2017 and 2018, which indicated that the drought resistance of *SiMYB56* transgenic rice was higher than that of wild-type controls during the reproductive stage in the field. Moreover, we found that the increased yield for transgenic rice plants was mainly attributed to higher values of panicles per plant and panicle length which is also well reflected in the phenotype (Figures 6A,B and Supplementary Figure S6).

### *SiMYB56* Overexpression Up-Regulated Lignin Biosynthesis Related Gene Expression Under Drought Conditions

**FIGURE 7:**
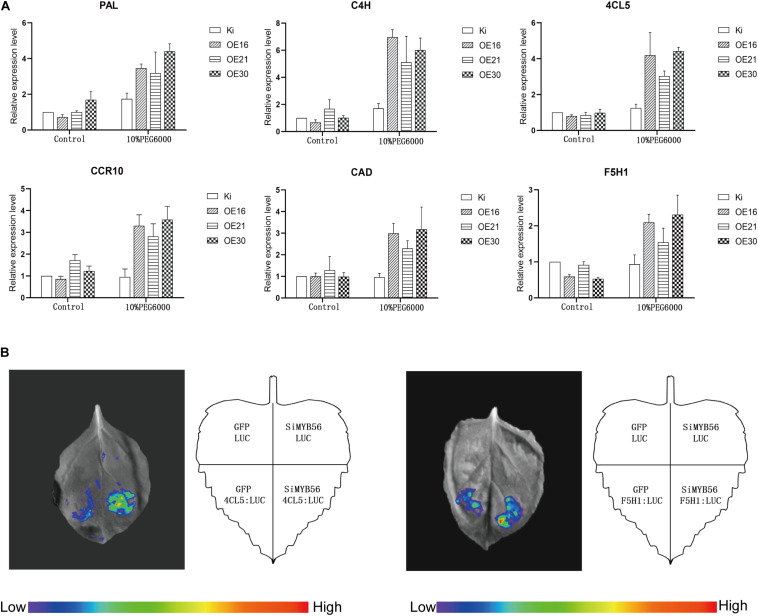
*SiMYB56* activates the expression of lignin synthesis related genes under drought conditions. **(A)** Expression analysis of lignin biosynthesis related genes in wild-type controls and transgenic rice plants. Entire 2-week-old *SiMYB56*-overexpressing and wild-type seedlings, growing under normal condition and 10% PEG6000 treatment, were harvested for RNA isolation. Transcript level was quantified by qRT-PCR. Values shown are means ± SD (*n* = 3). **(B)** Transcriptional activity assays in *Nicotiana benthamiana* leaves. SiMYB56 can activate the transcription of 4CL5 and F5H1. Four-week-old *Nicotiana benthamiana* plants were prepared for *Agrobacterium*-mediated transient expression, and generally the third through fifth leaf of each plant were analyzed. In each analysis, five independent *Nicotiana benthamiana* leaves were infiltrated and analyzed, and totally three biological replications were performed with quantification.

To elucidate the molecular mechanism of *SiMYB56* transgenic rice enhanced drought tolerance, the expression of several lignin biosynthesis related genes, including *PAL*, *4CL*, *C4H*, *CCR*, *CAD*, and *F5H* (Minami et al., 1989; Yang et al., 2005; Kawasaki et al., 2006; Zhang et al., 2006; Gui et al., 2011; Takeda et al., 2017), were measured with quantitative real-time PCR. The results showed that there was no significant expression difference for six lignin biosynthesis related genes under normal conditions; whereas, under drought conditions, the expression of the six genes was 2–8 times higher in transgenic rice plants than in wild-type controls (Figure 7A). Next, to explore the regulatory effect of co-expressed *SiMYB56* on transcription of *PAL*, *4CL5*, *C4H*, *CCR10*, *CAD*, and *F5H1*, Transcriptional activity assays were performed in *Nicotiana benthamiana* leaves with the promoters of these genes fused with a LUC reporter gene (PAL_pro_: LUC, 4CL5_pro_: LUC, C4H_pro_: LUC, CCR10_pro_: LUC, CAD_pro_: LUC, F5H1_pro_: LUC). Compared with empty vector (LUC), results showed that co-expression of 35S: SiMYB56 markedly increased the LUC expression driven by two promoters: *4CL5* and *F5H1* (Figure 7B). These results suggested that *SiMYB56* overexpression can increase the lignin content of transgenic plants by activating key lignin biosynthesis enzymes.

### Overexpression of *SiMYB56* Significantly Improves ABA Accumulation in Transgenic Rice Seeds and Activates the Expression of ABA Signaling Pathway Related Genes Under Drought Conditions

**FIGURE 8:**
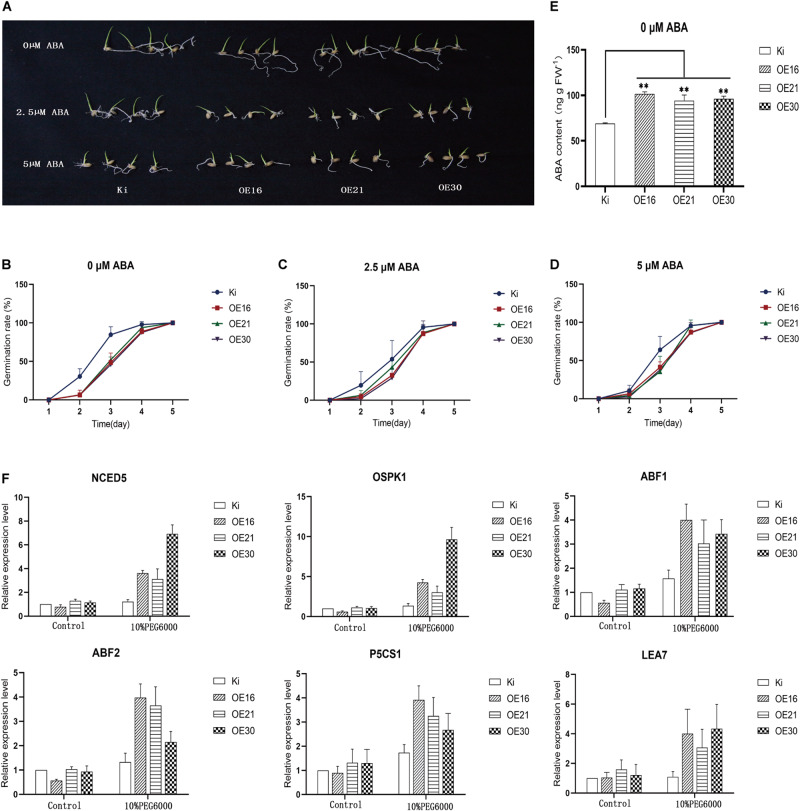
Overexpression of *SiMYB56* significantly improves ABA accumulation in transgenic rice seeds and activates the expression of ABA signaling pathway related genes under drought condition. **(A)** Seeds of wild-type controls and transgenic rice plants were germinated on plates containing different ABA concentrations. Germination rates of SiMYB56-overexpressing and wide-type seeds under 0 μM **(B)**, 2.5 μM **(C)**, and 5 μM **(D)** ABA treatments (*n* = 3, 16 seeds in each replicate). **(E)** ABA content of sprouts from germinated seeds of transgenic rice plants and wide-type controls under 0 μM ABA treatments. Values shown are means ± SD (*n* = 3). **(F)** Expression analysis of ABA signaling pathway related genes in wild-type controls and transgenic rice plants. Entire 2-week-old *SiMYB56-*overexpressing and wild-type seedlings, grown under normal condition and 10% PEG6000 treatments, were harvested for RNA isolation. Transcript level was quantified by qRT-PCR. Values shown are means ± SD (*n* = 3).

Abscisic acid can inhibit seed germination (Finkelstein et al., 2008). In order to explain the reduced seed germination rate of transgenic rice plants compared with wild-type controls, we analyzed seed germination rates of transgenic rice and wild-type seeds at different ABA concentrations (Figure 8A). Results showed that under normal conditions the germination rate of transgenic rice seeds was indeed lower than that of wild-type seeds (Figure 8B), while with 2.5 μM (Figure 8C) and 5 μM ABA (Figure 8D) the germination rate difference between transgenic rice and wild-type seeds narrowed. Then, we determined the endogenous ABA content in the germinated seeds, and results showed that germinated seeds of transgenic rice plants had a higher content than those of wild-type controls (Figure 8E), which suggests that *SiMYB56* could promote ABA synthesis in transgenic rice seeds.

Abscisic acid signaling pathway is important for plant response to drought stress (Yoshida et al., 2014), and *SiMYB56* overexpression led to ABA accumulation. Therefore, we speculated that *SiMYB56* could affect ABA signaling as another means of improving plant drought resistance. In order to test this hypothesis, we analyzed the expression of ABA synthesis related genes (*NCED5*), ABA signal transduction related genes (*ABIL2*, *OSPK1*, *ABF1*, *ABF2*, *bZIP23*), and ABA response related genes (*P5CS1*, *LEA7*) in transgenic rice plants and wild-type controls under both normal and drought conditions (Figure 8F). Results showed that *SiMYB56* enhanced ABA synthesis under drought conditions and activated the ABA signaling pathway, contributing to the enhanced drought tolerance of transgenic rice.

## Discussion

**FIGURE 9:**
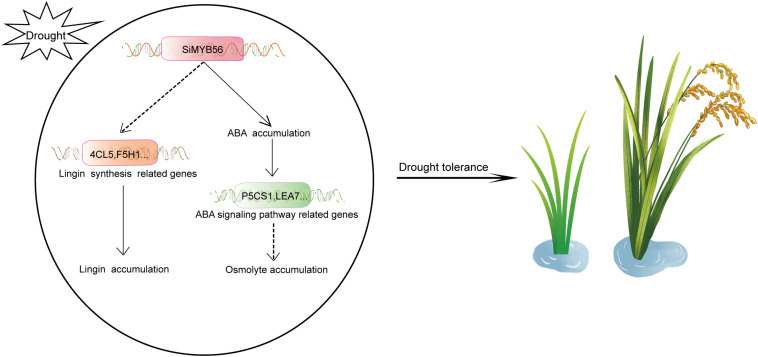
A proposed model for the role of *SiMYB56* in drought tolerance regulation. Under drought stress, *SiMYB56* expression is up-regulated, activating the expression of lignin synthesis related genes, such as *4CL5* and *F5H1*, which results in increased lignin content and subsequent drought tolerance. Up-regulation of *SiMYB56* also increased ABA accumulation and upregulates ABA responsive genes, such as *P5CS1* and *LEA7*, leading to osmolyte accumulation that supplements enhanced drought tolerance provided by increased lignin content.

In this study, we demonstrated that *SiMYB56* may enhance transgenic rice drought resistance both in greenhouse and field-drought conditions by activating the expression of lignin biosynthesis related genes lead to lignin accumulation (Figure 9). These results indicate that *SiMYB56* can advantageously be used to improve the drought resistance of field-grown gramineous crops. Gramineous crops are the main food source for the world’s population. With the aggravation of greenhouse gas effects and frequent drought, it is important to ensure global food security by improving the drought resistance of gramineous crops. Foxtail millet has a very high drought resistance, and regulation of lignin biosynthesis is an important part of that resistance. Increased lignification is a common response to biotic and abiotic stress (Moura et al., 2010). Studies have shown that many types of transcription factor improve plant drought resistance by regulating lignin biosynthesis. In rice, overexpression of *OsTF1L*, a rice HD-Zip transcription factor, promoted lignin biosynthesis and stomatal closure, which thereby improved drought tolerance (Bang et al., 2019). *OsERF71*, an AP2/ERF transcription factor, can modulate downstream genes, including general stress inducible genes, cell wall-associated genes, and lignin biosynthesis genes, further contributing to improved drought resistance (Lee et al., 2016). In white birch (*Betula platyphylla*), *BpNAC012* increased salt and osmotic stress tolerance by regulating abiotic stress-responsive downstream genes, including D-1-pyrroline-5-carboxylate synthetase, superoxide dismutase, and peroxidase, as well as secondary wall-associated downstream genes (Hu et al., 2019). These results show that regulation of plant drought resistance by mediating lignin biosynthesis is a highly conservative process in plants and should be a future target of plant drought resistance improvement via molecular marker assisted breeding.

In addition to lignin accumulation, drought stress also triggers a series of stress-response pathways in plants (Zhu, 2002). Under drought stress conditions, numerous stress response and tolerance genes are induced, and ABA, a key plant stress-signaling hormone, accumulates (Tuteja, 2007). ABA controls plant stress response at multiple layers of regulation. These include: (1) transcriptional response, including interactions of core transcription factors that are regulated by ABA and other plant hormones; and (2) regulation of ABA metabolism and transport, with posttranscriptional and posttranslational regulation that remains a hidden and poorly recognized aspect of stress signaling (Daszkowska-Golec, 2016). Through a variety of signal transduction, ABA eventually triggers a series of plant physiological changes, such as stomatal closure, proline synthesis, and ROS scavenging (Stewart and Voetberg, 1985; Jiang and Zhang, 2001; Eisenach et al., 2017). These physiological changes improve plant drought resistance. In our study, *SiMYB56* was shown to promote ABA synthesis, and quantitative real-time PCR indicated that *SiMYB56* can activate a series of ABA signaling pathway related genes under drought conditions, including a proline synthesis gene (*P5CS1*) and a late embryogenesis abundant protein gene (*LEA7*). This result indicate that *SiMYB56* may lead to osmolyte accumulation by activating ABA signaling (Figure 9), which in cooperation with lignin deposition improves transgenic rice drought resistance. ABA can rapidly accumulate in plants under drought stress to confer drought resistance, while lignin deposition mainly functions to relieve long-term drought stress (Terzİ et al., 2013; Geng et al., 2018; Zhang et al., 2018). Our study showed that *SiMYB56* may coordinate these two processes to improve rice drought resistance throughout a plant’s lifecycle.

High lignin content affects the degradation and utilization of straw (Chen et al., 2015), and excessive ABA content inhibits normal plant growth (Chen et al., 2020). However, in phenotypic experiments, it was found that there was no significant difference in lignin content between transgenic plant lines and wild-type plants grown under normal conditions, and *SiMYB56* overexpression did not affect rice growth under normal conditions. Quantitative real-time PCR also showed that the expression of genes related to lignin and ABA synthesis was up-regulated only under drought conditions, which means *SiMYB56* overexpression can only activate lignin and ABA synthesis under specific conditions. The mechanism by which *SiMYB56* functions under drought stress needs to be further explored, but it seems clear that *SiMYB56* overexpression does not affect normal plant growth.

## Accession Numbers

Sequence data from this article can be found in the Phytozome database under the following accession numbers: Seita.5G043900 (SiMYB56), LOC_Os02g41650 (PAL), LOC_Os08g34790 (4CL5), LOC_Os02g26770 (C4H), LOC_Os02g56700 (CCR10), LOC_Os08g43550 (CAD), LOC_Os10g36848 (F5H1), LOC_Os12g42280 (NCED5), LOC_Os05g51510 (ABIL2), LOC_Os02g34600 (OSPK1), LOC_Os01g64730 (ABF1), LOC_Os06g10880 (ABF2), LOC_Os02g52780 (bZIP23), LOC_Os05g38150 (P5CS1), and LOC_Os03g62620 (LEA7).

## Data Availability Statement

All datasets presented in this study are included in the article/Supplementary Material.

## Author Contributions

WX designed and performed the experiments and wrote the manuscript. WT helped with the experiments. CW contributed to the implementation of the study. LG helped to clone the *SiMYB56* gene. JS performed the field experiments. XQ and ZH contributed the valuable discussion. JC, YZ, and ZX provided instruction for the experiments. MC coordinated the project, conceived and designed the experiments, and edited the manuscript. Y-ZM coordinated the project and edited the manuscript. All authors have read and approved the final manuscript.

## Conflict of Interest

The authors declare that the research was conducted in the absence of any commercial or financial relationships that could be construed as a potential conflict of interest.

## Footnotes

1 https://www.arabidopsis.org/

2 https://phytozome.jgi.doe.gov/pz/portal.html

3 http://bioinformatics.psb.ugent.be/webtools/plantcare/html/
