# Supplementary material for: SiMYB56 Confers Drought Stress Tolerance in Transgenic Rice by Regulating Lignin Biosynthesis and ABA Signaling Pathway
Source: Front Plant Sci. 2020 Jun 18;11:785. doi: 10.3389/fpls.2020.00785 (PMC7314972; doi:10.3389/fpls.2020.00785)
Supplement: Supplementary file 1 [file Data_Sheet_1.PDF]

Supplementary Material

Supplementary Figures

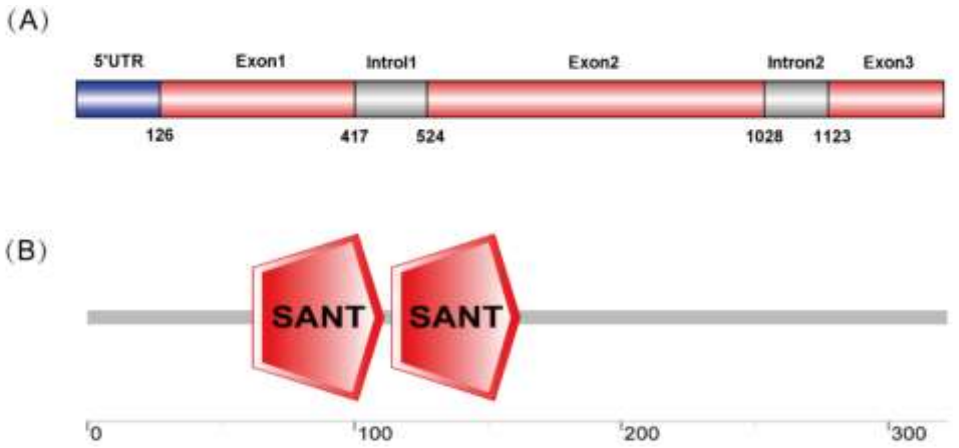

Supplementary Figure 1. Gene and protein structure of *SiMYB56.* (A) Gene structure of *SiMYB56*. (B) Protein structure of *SiMYB56*.

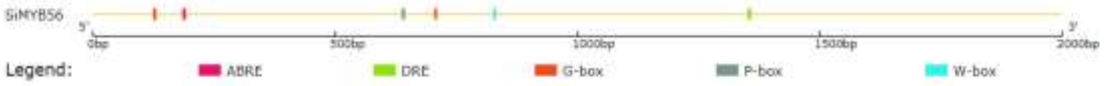

Supplementary Figure 2. *Cis*-acting elements in the promoter of *SiMYB56*.

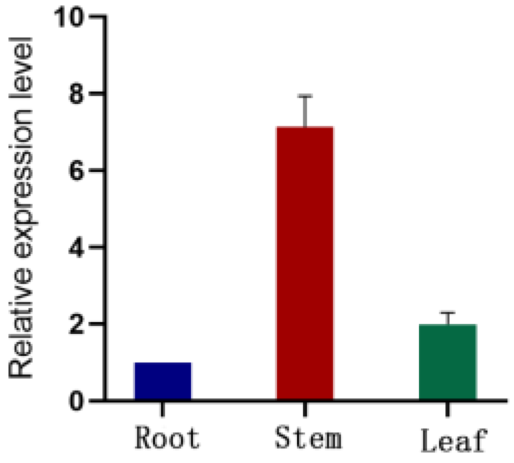

Supplementary Figure 3. Relative *SiMYB56* expression levels in different foxtail millet tissues. All *SiMYB56* expression levels were determined in triplicate by qRT-PCR. Values shown are means ± SD.

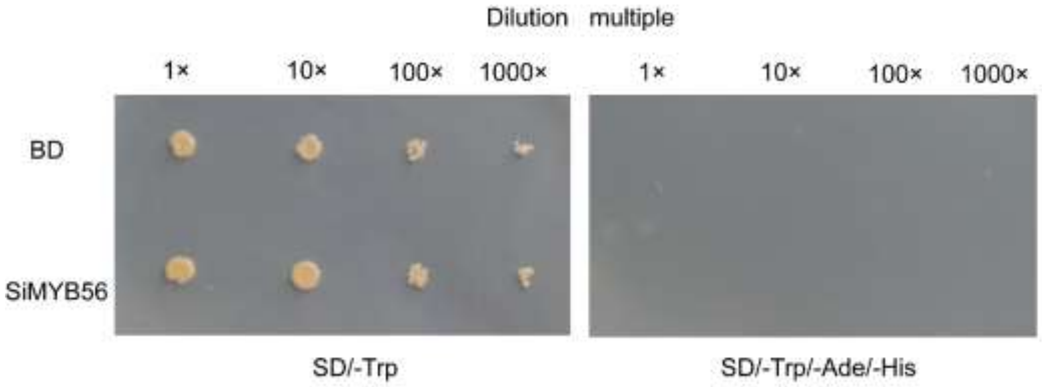

Supplementary Figure 4. Transcription activation activity of SiMYB56. (A) Transcription activation assay of SiMYB56 protein in yeast. Fusion vectors pGBKT7-SiMYB56 (SiMYB56-BD) and negative control pGBKT7 vector (BD) were transformed into yeast strain AH109, separately. Transformants were examined on SD/-Trp and SD/-Trp/-His/- Ade/.).

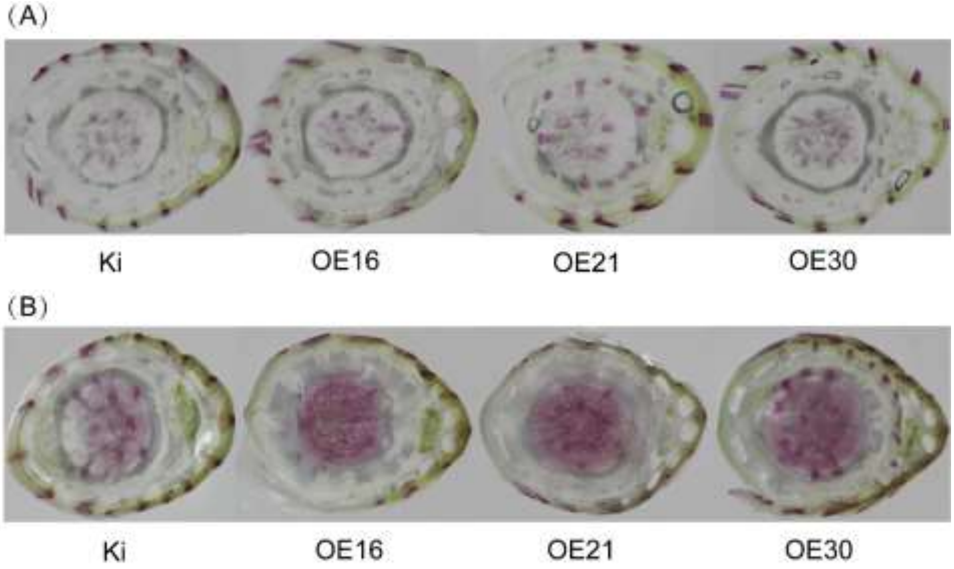

Supplementary Figure 5. Phloroglucinol staining for lignin in the leaves of transgenic rice plants and wild-type controls. (A) Phloroglucinol staining for lignin in the cross-sections taken from the leaves of transgenic rice plants and wild-type controls under normal condition. (B) Phloroglucinol staining for lignin in the cross-sections taken from the leaves of transgenic rice plants and wild-type controls under 10% PEG6000 treatment.

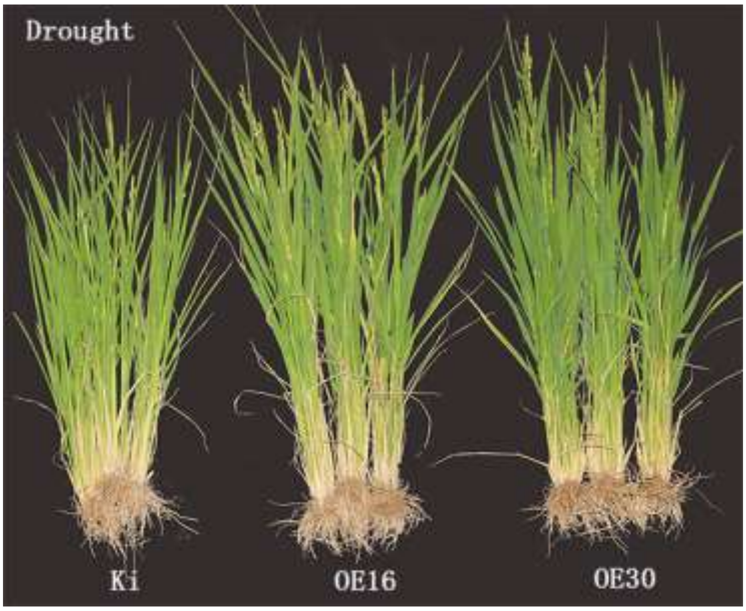

Supplementary Figure 6. Phenotype of transgenic rice plants and wild-type controls grown under field-drought condition in year 2018.

Supplementary Tables

Supplementary Table 1. Primer sequences used in this study

Primer name	Sequence
SiMYB56-F	TCATGTCGCACCAACGCCGAG
SiMYB56-R	ATGGTTTGTTTCTCCGGCCGTG
62-BD-F	ATCCCCCGGGCTGCAG ATGAAGCTACTGT
62-BD-R	TATCGAATTCCTGCAG CGATACAGTC
62-BD-SiMYB56-F	ATCCCCCGGGCTGCAGATGAAGCTACTGTC
62-BD-SiMYB56-R	TATCGAATTCCTGCAGTCATGTCGCACCAAC
BD-SiMYB56-F	AGGAGGACCTGCATATGATGGTTTGTTTCT
BD-SiMYB56-R	GCCTCCATGGCCATATGTCATGTCGCAC
GFP-SiMYB56-F	TATCTCTAGAGGATCCATGGTTTGTTTCTCC
GFP-SiMYB56-R	TGCTCACCATGGATCCTGTCGCACCAAC
pMWB014- SiMYB56-F	GACCGATCTGGATCC ATGGTTTGTTTCTCC
pMWB014- SiMYB56-R	TAGTAAGCCGGATCCTCATGTCGCA
1302-SiMYB56-F	GGGACTCTTGACCATGATGGTTTGTTTCTCC
1302-SiMYB56-R	TCAGATCTACCCATGGTGTCGCACCAAC
0800-4CL5-F	GCAGCCCGGGGGATCCAGTTTGAAGAAATTG
0800-4CL5-R	TAGAACTAGTGGATCCAGGAGTGGAAGCA
0800-F5H1-F	GCAGCCCGGGGGATCCTGGATAGTCCATTTG
0800-F5H1-R	TAGAACTAGTGGATCCAGAGTTGGGAGGAGT
Test-F	GCCCTGCCTTCATACGCTATTT
Test-R	CAAGACCGGCAACAGGATTCA
Siactin-F	GGCAAACAGGGAGAAGATGA
Siactin-R	GAGGTTGTCGGTAAGGTCACG
Osactin-F	CCTTCAACACCCCTGCTATG
Osactin-R	CAATGCCAGGGAACATAGTG
RT-SiMYB56-F	CTCTGTATCCGTTCCGCTTCC
RT-SiMYB56-R	GCTAATCTCCTCTGGGTCCTCTA
RT-PAL-F	TACAACAACGGGCTTCCTTC
RT-PAL-R	TGAGCTTCAGGATGTCGATG
RT-4CL5-F	GCAAGGAGCTTCAGGACATC
RT-4CL5-R	TTTCCCCTGATGCAAATCTC
RT-C4H-F	TGGTGAGGAGCTTCGAGATG
RT-C4H-R	TGAGTTCAGGCAGAGATGGG
RT-CCR10-F	TTGTCACGGTGGCACAACAG
RT-CCR10-R	ATATGCCGCCGCTGTCATGT
RT-CAD-F	TTGTCACGGTGGCACAACAG
RT-CAD-R	ATATGCCGCCGCTGTCATGT
RT-F5H1-F	GTGTGGTGTGTCATCCATGG
RT-F5H1-R	CGCATGATTAGGACGGCC
RT-NCED5-F	GACTGCTTCTGCTTCCACCT
RT-NCED5-R	TCGTTGAAGATGGAGTCGGC

RT-OSPK1-F	TTCAAGCTTTGCTGTTGCCG
RT-OSPK1-R	ATGCGACATCTCATCACCCC
RT-ABF1-F	GTCGTGGCACCAAACAGAGA
RT-ABF1-R	GGGAGAGGAAGCAGGCCTAT
RT-ABF2-F	GGCATGCCATCTGTGTTTGTT
RT-ABF2-R	GACAGCAACTTCGTCTCGCA
RT-P5CS1-F	ATTGCGGCATTCCGATGGAT
RT-P5CS1-R	CGGCGATATGTCGTAGCATGA
RT-LEA7-F	GTTCCACAGGTTCTCCTTCG
RT-LEA7-R	GCCGAGCCACATCTCATACT
